# “For those who don’t cry at night”: exploring narratives of integrative medicine practitioners treating hospital personnel during war

**DOI:** 10.3389/fmed.2025.1583444

**Published:** 2025-06-02

**Authors:** Elad Schiff, Eran Ben-Arye, Gali Stoffman, Ariela Popper-Giveon, Yael Keshet, Avi Goldberg, Chezy Levy, Ohad Hochman, Sameer Kassem, Samuel Attias

**Affiliations:** ^1^Rappaport Faculty of Medicine, Technion-Israel Institute of Technology, Haifa, Israel; ^2^Department of Internal Medicine B, Bnai Zion Medical Center, Haifa, Israel; ^3^Integrative and Complementary Medicine Service, Bnai Zion Medical Center, Haifa, Israel; ^4^Integrative Oncology Program, Oncology Service, Lin and Carmel Medical Center, Haifa, Israel; ^5^Integrative In-patient Services, Barzilai Medical Center, Ashqelon, Israel; ^6^David Yellin Academic College, Jerusalem, Israel; ^7^Western Galilee Academic College, Acre, Israel; ^8^Carmel Medical Center, Haifa, Israel; ^9^Barzilai Medical Center, Ashkelon, Israel; ^10^Bnai Zion Medical Center, Haifa, Israel

**Keywords:** integrative medicine, complementary medicine, hospital, war, acute stress, healthcare providers, resilience

## Abstract

**Background:**

Promoting the resilience of healthcare providers (HCP) is crucial during peaceful times and even more during national crisis. The outbreak of the war in Israel, Gaza strip, and Lebanon in October 2023 prompted the establishment of integrative medicine resilience clinics (IMRC) in three hospitals; incorporating evidence based integrative medicine (IM) modalities, for reducing emotional and physical concerns among HCP. Our objective was to explore the impact of the IMRC through narratives of IM practitioners who provided the treatments. To explore the impact of the IMRC through narratives of IM practitioners who provided the treatments.

**Methods:**

Qualitative narrative research was based on in-depth interviews with 16 IM practitioners from IMRC’s in three hospitals.

**Results:**

The interviewees’ narratives revealed four spheres where the IMRC’s contribution is suggested: 1- IM practitioners conceived their work to be effective in improving HCP wellbeing; 2- they felt that HCP functioned more effectively and provided better patient care; 3- practitioners described feeling meaningful, and acknowledged in the healthcare organization; 4- the positive impact of IM on HCP and administrators, positions them as potential advocates for IM in public health.

**Conclusion:**

IMRC for hospital HCP may have an important role in maintaining HCP resilience during wartime. These effects may also have ramifications on the recognition of the role of IM in public health during crisis and everyday times.

## Background

National crises, including wars, impose immense stress on healthcare providers (HCPs), leading to emotional exhaustion, secondary traumatic stress, acute stress disorder, and burnout. The psychological toll on HCPs during such periods can manifest through various health issues, including sleep disturbances, cardiovascular problems, aggressive behaviors, mental and physical fatigue, chronic pain, and even post-traumatic stress syndrome (1). This raises significant concerns about the well-being of HCPs, particularly in war-torn regions where the demands of their roles intensify dramatically.

A UK National Health System report titled “Healthy Staff, Better Care for Patients” underscores that the well-being of healthcare staff is intricately linked to reduced sick days, enhanced workforce efficiency, and ultimately, improved patient care. The report recommends increasing awareness of mental health issues among HCPs and implementing accessible interventions to promote good mental health (2). Traditionally, clinical practices have relied heavily on medications to address stress-related symptoms. However, the potential for long-term side effects from these medications underscores the necessity for non-pharmacological therapies, including the integration of complementary medicine modalities.

Significant evidence indicates that mind–body practices—such as yoga, massage, meditation, and mindfulness interventions—can effectively alleviate stress-related symptoms and consistently reduce subjective stress among HCPs (3–6). A comprehensive review by Balbinot and Bordignon (7) highlighted the effectiveness of integrative medicine (IM) approaches, including auricular acupuncture, music therapy, and mindfulness practices in preventing and managing stress and burnout among healthcare professionals.

In the aftermath of the Gaza and Lebanon-Israel war outbreak in October 2023, the immense pressures faced by Israeli HCPs prompted the establishment of Integrative Medicine Resilience Clinics (IMRC) across three medical centers. These clinics integrated complementary medicine modalities that have demonstrated effectiveness and safety regarding acute stress and burnout (3–6). Initiated by the hospitals’ IM practitioners and supported by medical and nursing administrations, these clinics aimed to alleviate war-related emotional and physical concerns among HCPs. The IM modalities offered included acupuncture, reflexology, shiatsu, acupressure, osteopathy, and guided imagery, paralleling treatments used for daily patient care in each center.

IM practitioners underwent specialized training co-designed by leading IM practitioners and trauma psychologists, focusing on alleviating acute stress disorder based on current research and field experience. The IMRC operated during morning hours to ensure accessibility for HCPs during their work shifts. Supported by hospital administrations, HCPs received notifications and were encouraged to utilize these services through referrals generated by department directors or internal hospital communications (e.g., emails and intranet). Over the course of 3–4 months, the IMRC across the three medical centers treated 532 HCPs, offering a vital lifeline during a time of unprecedented stress. The number of treatments per HCPs varied between the three centers from 1 to 3 on average. Recurring treatments were provided on a weekly basis.

As healthcare organizations increasingly integrate IM services—not only for patients but also for prioritizing staff well-being—there is an urgent need to understand the experiences of the providers delivering these interventions. Limited research suggests that practitioners providing support may grapple with uncertainties regarding their abilities, face blurred professional boundaries, experience vicarious trauma, and encounter ethical dilemmas (8). These complexities underscore the necessity of exploring the experiences and perceptions of IM practitioners in wartime settings.

Understanding the motivations, challenges, and needs of IM practitioners can inform the effective development, implementation, and sustainability of support programs for highly stressed HCPs. Consequently, our research objective was to gain deeper insights into how IM practitioners perceive their role and impact in the unique context of a hospital-based IMRC for HCPs operating during wartime.

## Methods

This research employed a qualitative narrative research design to explore the experiences of IM practitioners. Between January and February 2024, three months following the launch of the IMRC initiative, we conducted semi-structured in-depth interviews with a study population comprising 16 practitioners employed by the IM services in three public hospitals in Israel. One of the hospitals (Barzilai) is situated only 7 km north of the Gaza border and has experienced direct missile fire. Many HCPs at this hospital resided in areas under active conflict or had been evacuated from their homes. The other two hospitals (Carmel and Bnai Zion) are located approximately 45 km from the Lebanon frontline, where many staff faced missile threats or treated patients evacuated from the northern border. The IM practitioners interviewed participated at the IMRC’s that were operated in the three hospitals.

The number of interviews conducted allowed saturation to be achieved: both code saturation -when researchers have “heard it all,” and meaning saturation-when researchers have understood it all (9). Research questions were designed to explore how IM practitioners experienced their role in the IMRC during wartime and their perceptions of the initiative’s impact on both themselves and their HCP clients. Participants were informed about the research objectives, and assured anonymity, with oral informed consent obtained prior to the interviews. The interviews were audio recorded, and subsequently transcribed verbatim by a professional company to ensure accuracy.

A thematic analysis (10) was conducted to identify key themes from the qualitative data. The analysis process included six stages: 1. reading and re-reading the data and noting initial ideas, conducted independently by two experienced qualitative researchers (YK, APG) to enhance trustworthiness. The interview scripts were coded line by line by APG to generate themes. Independently, YK also generated themes, and a consensus on the coding framework was reached. 2. After establishing the coding scheme, APG systematically coded interesting features of the data, collating relevant data for each code; 3. Gathering codes into potential themes; 4. Generating a thematic “map” of the analysis; 5. Refining each theme to reveal the overall story illustrated by the analysis; and 6. Preparing a scholarly report of the analysis. Atlas.ti v9 software was utilized develop the coding schemas summarizing the topics and concepts emerging from the data. Following Saldaña’s (11), we applied two cycles of analysis. The first cycle coding, summarized in Table 1, includes codes initially identified in and assigned to the data, relating to its content, and supporting citations from the interviews. The second cycle coding, described in the following Result section, resulted from the first cycle codes and refers to more abstract categories.

**Table 1:** Themes and associated citations from integrative medicine practitioners.

Sphere	Theme	Citations
HCP Sphere	Treatment effectiveness	There are those who say ‘Yes, there [during the IM intervention] I feel a bit detached from everything. I… feel calmer’… Some say that this effect continues overnight, they sleep better that night. Many point this out. Many point out that they leave with more energy, they can return to work better. It recharges them. (Shai)
		[HCP described] how it changed for them, how they slept better at night, that they did not sleep and now suddenly the first night they slept… Their level of fear decreased, as if they have tools to cope. (Dana)
		Acupuncture or shiatsu or reflexology… there is no dynamic discourse in them. There is silence and inwardness… which is good and right especially in acute stress. It does allow for a… very very calm feeling. It allows to sleep for a moment, rest, detach from thoughts. (Nir)
	Dominant symptoms at early phase of IMRC	The hardest psychologically [time was] in the first week, really in the first week that we treated. There was a woman who came with ((stops breathing)). Wow, it was really bad, and I was so happy that I managed to lift her up… She was so overwhelmed… you ask her how she is and she immediately… starts crying… you do not know why, you suddenly have a high pulse, but when you put a word to it, ‘I’m anxious’, okay, then it’s fine. And when they tell you… it’s a physiological phenomenon, it’s a normal reaction to an abnormal situation… it helps us feel okay… [it gives] legitimacy to feel that way. (Bella)
	Evolution of complaints over time	A doctor came with… a complaint of stiff shoulders and a sore neck. I do not know why, I decided not to treat her orthopedically… I referred more to the condition of the internal organs (a physiological concept in traditional Chinese medicine), which mainly showed great anxiety and stress… The next day she saw me at… the hospital and told me ‘Listen, it really helped me and I feel my shoulders are less stiff. I’m less tense. I slept well at night’. (Nir)
		“There is a tendency for men to complain only physical complaints… they are men, strong people, and do not cry at night, but slowly, slowly as you treat them… in the end I would always ask ‘what did you get from the treatment?’, oh, it’s relaxing, oh it’s like things they did not complain about before ((smiling)) (Gila)
	Perception of treatment outcomes	People came on their own initiative and people came following others, who felt it did them good. Really like a snowball effect… People… reported to their superiors that treatment helped them, they are coming back. Those same managers send them, during working time… it’s critical, in hospital the morning hours are the hours of treatments, the busiest hours. And yes… people come and that says it all. (Shai)
		Nurses come, doctors come. More and more. Yes… I think they… hear kind of… word of mouth … the advantages… [They] enjoy this service and tell their friends, so they allow themselves to take more time and come. (Maya)
IM practitioner sphere	Sense of mission and contribution	I’ve been practicing Chinese medicine for twenty years. It’s not new to me that it works. But still, the ability to put a few needles in a person in a very, very difficult situation, whose objective reality is terrible or who is experiencing a terrible experience, and you can bring them some relief, even if partial, even if for a period of days, until the next treatment. It never ceases to fill me with wonder and awe and gratitude, too, that I have such a tool. (Gabriella)
		We had a sense of mission even long before…we worked a lot together to promote our field, so there was also a great sense of mission there. And here too, there is a constant sense of mission. I think this sense of mission was greatly enhanced because there is feedback, because we see it in our indicators of Chinese medicine too, and also [because] the responses of those treated… Since the need is growing, awareness is growing too. (Nadav)
	Increase in self-esteem of providers	Since this clinic started, I feel much more familiar in the hospital. That is, people talk to me much more and involve me much more, I give many more “high fives.” I’m relatively new because I’ve… only been here for two years. In quite a large system that’s considered new. So I was very excited about this. (Nir)
	Treatment effects on oneself and self-care	When I treat, I feel very centered, it’s like a kind of meditation for me. When I enter it, I’m less occupied with my own things: with my son who is fighting in the north frontier, and with my son-in-law who is [fighting] in Gaza… I’m in the here and now… There’s something in the treatments, like an exchange with the patients, kind of symbiosis…and often after treatments I feel better. (Aya)
		I practice the things I learnt by myself how to do, various ways to deal with things, such as learning positive thinking. The same things that I suggest for my patients - I do myself as well (Dorin)
		I pay attention to myself. I admit I breath a lot after [treatments]; I try to walk, I take care of myself… After [treatments] I do not know what, I go to the pool… I meditate or whatever (Dana)
		When I treat patients I feel organized, collected. Treating is like a kind of meditation for me. I get into it, and I’m less busy with my own things… There’s something in giving treatments that resembles metabolism with the patients, some kind of symbiosis with them… often after treatments I feel better (Dana)
		First there was fear and then it changed: ‘I’m strong, I can get through this, I have something important that I can help people with, and I can support and I can give’, so that kept me going on. It gave me a feeling that I’m part of the people who save [others]. Like a soldier who protects on the fences, I’m also a soldier in the hospital who help people. It gave me strength to feel that I have a tool, that I will not sit at home… It gave me the strength to leave home and come here… It gave me a feeling that yes, I have a very important role, I can help people. (Meir)

	Nature of treatments with avoidance of psychodynamic interactions	We’re not in the field of mental health… and… the complains are mostly mental. We treat mostly in the… physical field. Although we influence the mental part, a lot, but we know it is connected, body–mind… We went through some training in the field of… acute stress in these situations. And the recommendation was not to go into emotional therapy because it can very quickly trigger things that we do not… have the tools to deal with… Most of these situations, the emotional ones… have a physical expression… People express it either in… lack of sleep or in… lower back pain which expresses anxiety, neck, shoulder, physical expressions. Most of the time, as soon as these are taken care of… indirectly we can take care of it… without touching the event itself (Shai)
	Practitioners and the warrior metaphor	First there was fear and then it changed: ‘I’m strong, I can get through this, I have something important that I can help people with, and I can support and I can give’, so that kept me going on. It gave me a feeling that I’m part of the people who save [others]. Like a soldier who protects on the fences, I’m also a soldier in the hospital who help people. It gave me strength to feel that I have a tool, that I will not sit at home… It gave me the strength to leave home and come here… It gave me a feeling that yes, I have a very important role, I can help people. (Meir)
I felt as if … [though] I do not have a weapon… I’m in battle, as if in my mind I was hyper-arousal… It’s not fear but it’s some kind of feeling I have not experienced since the army… I have not experienced such vigilance and kind of action in a long time. (Nir)	
The hospital sphere	Perceiving the organization’s role in supporting its employees	I think it’s also a message from the hospital that they care about you. You’re doing it here during work hours… not on your own time. They allow you this, which is a very important message from the hospital. (Shai)
		It’s important on the level of the individual that is being helped and [it’s] important on the level of the system that knows… The system is very demanding and very difficult. You have to work so many shifts from this hour to that hour, you have to treat, you have to be at your best and so on. So suddenly there is some place to have compassion, to accept that the [workers are] not at their best right now, and we are helping them deal with it. We’re not just judging them and getting angry at them. (Bella)
	IMRC effect on employee patient care	Once the staff is more balanced, physically, mentally, emotionally, spiritually, they’ll be able to better contain the hospitalized patients, the sick, and give better service… On the micro level, the macro, the department, the hospital… [War] is an extreme situation… people are exposed to more stress… people are more frail now, physically and emotionally. (Dan)
		War impacts the well-being of the people, and in some way it also impacts their output, so in terms of a worthwhile investment I think it’s a very worthwhile investment for any organization to invest ((smiling)) in the well-being of its employees, so that their output will be as high as possible. (Saar)
	IMRC increasing visibility of practitioners in their institutions	There are many good things happening around it [the clinic]… awareness in the hospital has greatly increased and our… sector… we do not even have a position… We do not have a… defined place within the hospital. [Following the clinic] we started to take a place and demand recognition. (Shlomo)
		“This clinic is also important in terms of visibility in the hospital… You cannot ignore that… that we, we exist, we are here, we can help. (Shai)
The public healthcare system sphere	Similarity to crisis response during COVID-19	Even during Corona, which was just before I started working, there was also a clinic intending to support staff so I think it’s some kind of mechanism that keeps repeating itself. People expect the service to… operate in these situations… Hospital workers expect the… integrative medicine to enlist when there is some kind of crisis like we have been experiencing for the last five years. (Nir)
		The initiative started back in the Corona, when we treated the staff. We volunteered; we worked inside the department with patients and also with staff… and very quickly it became clear that it was actually the staff who needed it more… And when the war started then immediately [those] who worked with us during Corona said ‘wait, maybe we need to provide support here too, the staff is broken’. (Gila)
	Increasing accessibility of diverse populations to integrative treatments	You’re exposed to many from the hospital staff. Their experience with this thing, because it’s free, because it’s during working hours, it attracts even more to come… In my opinion it raised the reputation and importance of the service tremendously (Nir)
		[The exposure] creates more openness, more understanding of this field. There is a lot in it, it’s an area that is a bit vague for many conventional medicine staff members. Once they are exposed, they also ask concrete questions, they get some enrichment from this field, they understand more what it does and how it works and how it can help… The connection between the integrative medicine staff and the conventional staff creates more effective integration. (Dan)
		Many of them are not familiar with integrative medicine. [Once] they know what it is, it opens their minds, it’s a step on the way to integration… Those who come are young resident doctors, mostly young doctors… You could say they are more open to it. Maybe because they have already experienced it or heard about it. (Ruth)
The public healthcare system sphere	IMRC as an Archimedean point to leverage IM in the health system	And then some doctor came… who had a very good experience from the ward and wants to bring us there. That is, it does trickle down, it does trickle down. Not a lot, like we wanted, but… it does trickle down. (Bella)
		These people are ultimately the ones who also refer [patients] to us and call us for consultations. If they know the value of what we do, then it’s much easier for them to also refer patients for us later (Nadav)
		To publish research. What is it good for? I hope we’ll see it also in numbers. How it helped people, how this treatment helped them and then to take it to the national level… to budget hospitals, HMOs and integrative medicine. To budget as required… people here do not get a normal salary, okay?… young people… family men, it frustrates them. (Bella)

Quantitative data including questionnaires assessing acute stress reactions (National Stressful Events Survey Acute Stress Disorder Short Scale), and major concerns (Measure Yourself Concerns and Wellbeing), as well as physiological changes (using Heart Rate Variability), were also collected from HCPs. These quantitative outcomes will be published separately.

The article is consistent with Standards for Reporting Qualitative Research (SRQR). All the data of the study are available from the corresponding author. The study received approval from the Ethics Review Board (Helsinki Committee) at Barzilai University Medical Center (0093-23-BRZ) and Bnai Zion Medical Center (BNZ-0105-23), and was exempted from requiring IRB approval at Carmel Medical Center, ensuring compliance with ethical standards in research.

## Results

### Description of the study group

The interviewed IM practitioners included 9 women and 7 men, 15 Jews and 1 Arab, ranging in age from 32 to 61. Most had received their education in complementary medicine colleges in Israel, while some were trained in Germany, the USA, and China. Their specializations in IM included acupuncture, touch and movement therapies (Feldenkrais, Paula method, osteopathy, Chi Gong), and mind–body practices (guided imagery, mindfulness, etc.). Many of the IM practitioners had participated in IM interventions for HCPs in their hospitals during the COVID-19 pandemic (12).

Moreover, a significant majority of the practitioners (13 out of 16) attended a specialized course in November 2023 on managing acute stress disorder using IM treatments, provided by the Israeli Society for Complementary Medicine, within the Israeli Medical Association, in collaboration with leading trauma psychologists. This additional training equipped practitioners with valuable skills for addressing the unique challenges presented by wartime stress. The interviews lasted between 30 and 60 min.

The interviewees’ narratives revealed four spheres in which the IMRC’s impact was evident: HCPs, IM practitioners, the hospital, and the public healthcare system. Key citations from the practitioners associated with each theme are summarized in Table 1.

### HCP sphere

IM practitioners observed that HCPs reported a variety of emotional and physical concerns attributable to stress related to the war, including heightened anxiety, insomnia, headaches, and muscular pain. Some complaints represented new onsets due to the war, while others were exacerbations of pre-existing medical conditions. IM practitioners assessed their treatments as effective based on HCPs feedback indicating reduced fear and anxiety, improved focus, better sleep, and increased energy levels. They noted that relief from stress and anxiety following IM treatments was particularly evident among those seeking support at the war’s onset, when stress levels were exceedingly high.

Over time, the nature of the complaints treated with IM evolved. Initially, most HCPs reported anxiety and stress, but as the weeks progressed, complaints became more somatic, primarily involving musculoskeletal pain and relapses of pre-war health issues. The IM practitioners perceived the ongoing referral and attendance to IM treatments as an indication of positive treatment outcomes.

### IM practitioner sphere

The IM practitioners reported that the experience of providing treatment to the hospital personnel at war deeply affected them. First and foremost, they had the opportunity to care for, and help people during a national-scale crisis, shared also by themselves, which generated a strong sense of contribution. This was intensified by the positive feedback reported by HCPs.

The IM practitioners also reveal that their involvement in the war-related IM project filled them with a sense of mission, and reduced their own stress and anxiety. They shared how they avoid emotional exhaustion, and secondary traumatic stress.

Working in the IMRC enabled IM practitioners to engage with diverse healthcare professionals across various departments, which fostered a sense of professional recognition and enhanced their self-confidence within the hospital system.

IM practitioners highlighted that during treatments they avoided psychodynamic interactions, and focused on IM methods to relieve emotional distress, and provide a “grounding” (safe-place) experience.

The interaction with HCPs was perceived by IM practitioners to be different from their interactions with “regular” patients. Due to the nature of the IMRC, HCPs primarily received stress-focused, short-term treatments. Additionally, IM practitioners felt it was not appropriate to explore the deeper emotional-spiritual aspects with their colleagues, an approach they routinely take with their “regular” patients. The hierarchical relationships within the hospital and the potential for ongoing workplace interactions created professional boundaries that limited the therapeutic scope.

### The hospital sphere

IM practitioners perceived that the IMRC demonstrated hospital leadership’s recognition of integrative medicine’s significant role in maintaining HCPs wellbeing. This recognition was evidenced by the provision of dedicated space for the IMRC and by allowing employees to access treatments free of charge during working hours.

The IMRC was perceived to enhance patient care by improving HCP wellbeing, consequently leading to better focus, improved clinical communication, and reduced burnout—all of which are reported to positively affect patient care (2).

In addition, it was felt that HCPs positive experience may change their approach to integrative medicine and possibly turn them into ambassadors of the idea of integration in the hospital. This gave rise to the hope that more departments in the hospital will too implement IM treatments.

### The public healthcare system sphere

Some of the interviewed practitioners reflected on their experience in treating hospital personnel with IM in COVID-19 isolated departments during 2020–2021. Interviewees felt that their prompt response to the present war-related activity was made possible because “hearts were trained” in reducing HCPs burnout during COVID-19. Following the positive experience gained during the COVID-19 period, there was an expectation by the hospitals’ administrations and personnel that IM practitioners will provide support for HCPs in the current war crisis as well.

The IMRC’s influence was perceived to extend beyond the individual hospital settings to the national healthcare landscape. Practitioners expressed optimism regarding the potential for policy changes that might support the integration of IM practices into mainstream healthcare in Israel. They believed that positive outcomes reported by HCPs as well as the qualitative and quantitative research accompanying the project could serve as a catalyst for broader acceptance of IM in public health initiatives.

## Discussion

The present study sheds light on how IM practitioners perceive their work in a hospital-based IMRC during a national war crisis. There are only a handful of studies on providers’ perspectives of those attending to HCPs mental health during crisis such as the COVID-19 pandemic (13, 14, 25). Similar to these studies, although IM practitioners welcomed the opportunity to assist their colleagues, challenges such as taking care of their own health were significant.

Our study is, to the best of our knowledge, the first to explore how IM practitioners, a growing group within the healthcare system, reflect on their role in IM clinics during wartime. The findings corroborate previous research that emphasizes the effects of IM treatments during crises as perceived by IM practitioners (15). Interestingly, practitioners identified a dual nature in HCP complaints, highlighting both somatic and emotional challenges. Notably, HCPs seeking help for physical complaints frequently reported emotional relief following treatments. This aspect is particularly significant, given the reluctance many HCPs exhibit when it comes to disclosing mental distress (16, 17). The ability of IM practices to offer “grounding” i.e. a sense of safety and control in the midst of overwhelming emotions, appears particularly valuable in acute stress settings, fostering a sense of detachment from the chaos that surrounds HCP during war. Thus, the route of IM clinics during crisis, in contrast to psychological support clinics, offers HCPs a “safe way” to treatments for somatic complaints without the stigma of being emotionally vulnerable. Based on our qualitative analysis, hospital administrators should consider such a “hidden” benefit of IMRC in turbulent times, which has an advantage for some HCP over psychological stand- alone interventions.

For IM practitioners, involvement in the clinic has been profoundly gratifying and empowering. This aligns with findings from literature on the positive effects of meaningful work in healthcare, which can significantly enhance job satisfaction and personal well-being (18). Integration not only enhances patient care but also enriches IM practitioner’s sense of professionalism and belonging as a significant element of the medical system by validating their role and expertise in a biomedicine-dominated field.

It is interesting to note that IM practitioners did not report vicarious trauma, as described in other studies (19). This can be explained by minimal psychodynamic interactions during IM treatments entailing less immersion with negative experiences. Furthermore, the IM practitioners’ well-being practices may differ from mental health workers, making them less prone to such adversities.

In Israel, the government does not regulate IM practitioners, and IM services are not provided systematically in hospitals. Studies have shown that the most important factor influencing physicians’ referral to IM treatments is personal experience (20). The IMRC’s described here offered an outstanding opportunity for self-experiencing IM without prejudgment. How that may affect referral to IM and it’s assimilation in the medical establishment in more peaceful times is yet to be studied.

Building resilience in HCPs includes facilitating an adaptive attitude and behavior that allows one to remain psychologically healthy, or even to thrive after being exposed to stressful events (21). According to the interviews with the IM practitioners, the IMRC echoed toward that end by creating a silo for self-care within a stressful workplace. The hospital’s commitment to the well-being of its workers, expressed by providing IM treatments during working hours, sends a powerful message about its values and priorities, particularly in valuing staff health alongside patient care. This in turn is essential for maintaining high levels of engagement and productivity during times of crisis (22).

The IMRC has also played a pivotal role in promoting IM more broadly on both a national and international level. The double imprint of IMRC during the COVID-19 pandemic and the 2023–2024 war, provided within academic-governmental hospitals, is significant, and its outcomes were assessed within research and published in international peer reviewed journals (12, 15, 23, 24). This reflects the interest of the public as well as stakeholders in the issue of HCP burnout during catastrophes. We believe that the IMRC’s may serve as a catalyst for changing perceptions and increasing openness toward integrative approaches among medical professionals and stakeholders. This shift is crucial for the future of IM, potentially leading to more integrative, patient-centered care models in national health care.

### Limitations

This study was conducted in three hospitals in Israel, which may limit the generalizability of the findings to other healthcare settings. The hospital’s organizational culture, level of IM endorsement, and dedicated resources for HCPs support may not be readily available in other contexts. Furthermore, this study primarily captured the perspectives of IM practitioners themselves, without directly exploring the perceptions of the HCP who received the treatments and the hospital directors endorsing the IMRC. Future research should incorporate the viewpoints of HCPs and stakeholders to gain a more comprehensive understanding of the acceptability and effectiveness of IM interventions in enhancing resilience and well-being during crises.

## Conclusion

The IMRC’s represent a promising model for supporting the well-being of HCP during times of crisis, such as wartime. The implementation of such clinics may promote IM both on an institutional as well as on a national public health level. Further research is needed to assess the transferability of this IM-based model to diverse healthcare contexts.

## Data Availability

The raw data supporting the conclusions of this article will be made available by the authors, without undue reservation.

## References

[1] De Hert S. Burnout in health care workers: prevalence, impact and preventative strategies. Local Regional Anesthesia. (2020) 13:171–83. doi: 10.2147/LRA.S240564, PMID: 33149664 PMC7604257

[2] Department of Health and Social Care (2012). NHS health and well-being improvement framework. GOV.UK. Available online at: https://www.gov.uk/government/publications/nhs-health-well-being-improvement-framework

[3] Garland EL Gaylord SA Fredrickson BL. Positive reappraisal mediates the stress-reductive effects of mindfulness: an upward spiral process. Mindfulness. (2011) 2:59–67. doi: 10.1007/s12671-011-0043-8

[4] Kaushik M Jain A Agarwal P Joshi SD Parvez S. Role of yoga and meditation as complimentary therapeutic regime for stress-related neuropsychiatric disorders: utilization of brain waves activity as novel tool. Journal of evidence-based. Integ Med. (2020) 25:2515690X2094945. doi: 10.1177/2515690X20949451, PMID: 32985243 PMC7545749

[5] Morton ML Helminen EC Felver JC. A systematic review of mindfulness interventions on psychophysiological responses to acute stress. Mindfulness. (2020) 11:2039–54. doi: 10.1007/s12671-020-01386-7

[6] Zhang M Murphy B Cabanilla A Yidi C. Physical relaxation for occupational stress in healthcare workers: a systematic review and network meta-analysis of randomized controlled trials. J Occup Health. (2021) 63:e12243. doi: 10.1002/1348-9585.12243, PMID: 34235817 PMC8263904

[7] Balbinot MA Bordignon M. Strategies for management of stress and burnout among healthcare professionals in Brazil. Revista Brasileira de Medicina do Trabalho. (2022) 20:487–97. doi: 10.47626/1679-4435-2022-653, PMID: 36793460 PMC9904830

[8] Billings J Bloomfield M Greene T. Who helps the helpers? Healthcare Counsel Psychother J. (2021) 21:12–4. doi: 10.1186/s12913-021-06917-z, PMID: 34488733 PMC8419805

[9] Hennink MM Kaiser BN Marconi VC. Code saturation versus meaning saturation: how many interviews are enough? Qual Health Res. (2017) 27:591–608. doi: 10.1177/1049732316665344, PMID: 27670770 PMC9359070

[10] Braun V Clarke V. Using thematic analysis in psychology. Qual Res Psychol. (2006) 3:77–101. doi: 10.1191/1478088706qp063oa

[11] Saldaña J. The coding manual for qualitative researchers.3rd ed. Thousand Oaks (CA): SAGE Publications (2016).

[12] Ben-Arye E Zohar S Keshet Y Gressel O Samuels N Eden A. Sensing the lightness: a narrative analysis of an integrative medicine program for healthcare providers in the COVID-19 department. Support Care Cancer. (2022) 30:1419–26. doi: 10.1007/s00520-021-06546-6, PMID: 34528124 PMC8442644

[13] Billings J Biggs C Cing BCF Gkofa V Singleton D Bloomfield M. Experiences of mental health professionals supporting front-line health and social care workers during COVID-19: qualitative study. BJPsych Open. (2021) 7:70. doi: 10.1192/bjo.2021.29, PMID: 33752774 PMC8007934

[14] Rosen B Preisman M Read H Chaukos D Greenberg RA Jeffs L. Providers’ perspectives on implementing resilience coaching for healthcare workers during the COVID-19 pandemic. BMC Health Serv Res. (2022) 22:780. doi: 10.1186/s12913-022-08131-x, PMID: 35701756 PMC9194890

[15] Ben-Arye E Gressel O Samuels N Stein N Eden A Vagedes J. Complementary and integrative medicine intervention in front-line COVID-19 clinicians. BMJ Support Palliative Care. (2022) 14:e1192–200. doi: 10.1136/bmjspcare-2021-003333, PMID: 35383045

[16] Dyrbye LN West CP Sinsky CA Goeders LE Satele DV Shanafelt TD. Medical licensure questions and physician reluctance to seek care for mental health conditions. Mayo Cinic Proceedings. (2017) 92:1486–93. doi: 10.1016/j.mayocp.2017.06.020, PMID: 28982484

[17] Ng IK Goo TBC Al-Najjar S. Mental health stigma in the medical profession: where do we go from here? Cini Med. (2024) 24:100013. doi: 10.1016/j.clinme.2024.100013, PMID: 38382183 PMC11024831

[18] Adams JM. The value of worker well-being. Public Health Rep. (2019) 134:583–6. doi: 10.1177/0033354919878434, PMID: 31600480 PMC6832080

[19] Rosen B Preisman M Hunter J Maunder B. Applying psychotherapeutic principles to bolster resilience among health care workers during the COVID-19 pandemic. Am J Psychother. (2020) 73:144–8. doi: 10.1176/appi.psychotherapy.20200020, PMID: 32985915

[20] Berman BM Bausell RB Lee W. Use and referral patterns for 22 complementary and alternative medical therapies by members of the American College of Rheumatology: results of a national survey. Arch Intern Med. (2002) 162:766–70. doi: 10.1001/archinte.162.7.766, PMID: 11926849

[21] Sisto A Vicinanza F Campanozzi LL Ricci G Tartaglini D Tambone V. Towards a transversal definition of psychological resilience: A literature review. Medicina. (2019) 55:745. doi: 10.3390/medicina55110745 31744109 PMC6915594

[22] Raaper R Brown C. The Covid-19 pandemic and the dissolution of the university campus: implications for student support practice. J Professional Capital Community. (2020) 5:343–9. doi: 10.1108/JPCC-06-2020-0032

[23] Ben-Arye E Samuels N Kassem S. On being a stranger in a foreign land: providing integrative oncology therapies to COVID-19 medical professionals. Oncologist. (2022) 27:e973–5. doi: 10.1093/oncolo/oyac201, PMID: 36200861 PMC9732254

[24] Vagedes J Kassem S Gressel O Samuels N Eden A Ben-Arye E. Parasympathetic versus sympathetic changes in heart rate variability after a multimodal integrative medicine intervention for frontline COVID-19 personnel. Psychosom Med. (2023) 85:53–60. doi: 10.1097/PSY.0000000000001153, PMID: 36346679

[25] Liljestrand R Martin S. Stress and resilience among healthcare workers during the COVID-19 pandemic: consideration of case studies. Rehabil Nurs. (2021) 46:300–4. doi: 10.1097/RNJ.0000000000000344, PMID: 34469405 PMC8565454

